# Mediating effects of attitude on the relationship between knowledge and willingness to organ donation among nursing students

**DOI:** 10.3389/fpubh.2024.1370635

**Published:** 2024-04-09

**Authors:** Xiaohang Chen, Xin Zhou, Yan Xu, Li Pan, Peizheng Li, Wenjin Liang, Lv Jin, Chunhua Zhang, Chongxiang He

**Affiliations:** ^1^Zhongnan Hospital of Wuhan University, Institute of Hepatobiliary Diseases of Wuhan University, Transplant Center of Wuhan University, National Quality Control Center for Donated Organ Procurement, Hubei Key Laboratory of Medical Technology on Transplantation, Hubei Clinical Research Center for Natural Polymer Biological Liver, Hubei Engineering Center of Natural Polymer-based Medical Materials, Wuhan, China; ^2^Department of Biostatistics, School of Public Health, Wuhan University, Wuhan, China; ^3^Department of Nursing, Zhongnan Hospital of Wuhan University, Wuhan, China

**Keywords:** nursing students, organ donation, knowledge, attitude, willingness

## Abstract

**Background:**

The current rate of organ donation in China falls significantly below the global average and the actual demand. Nursing students play a crucial role in supporting and promoting social and public welfare activities. This study primary aims to analyze the levels of knowledge, attitudes, willingness toward organ donation, and attitudes toward death among nursing students, and investigate the mediating role of attitude in the relationship between knowledge and willingness. The secondary aims to identify factors that may influence the willingness.

**Methods:**

A convenience sample of nursing students completed online-administered questionnaires measuring the level of knowledge, attitudes, and willingness toward organ donation before and after clinical internship. Spearman correlation and mediation analyses were used for data analyses.

**Results:**

Before the clinical internship, there were 435 nursing students who had not yet obtained their degrees and were completing their clinical internships. After the internship, this number decreased to 323. The mean score for knowledge before and after the clinical internship (7.17 before and 7.22 after, with no significant difference), the attitude (4.58 before and 4.36 after, with significant difference), the willingness (12.41% before and 8.67% after, with significant difference), the Death Attitude Profile-Revised (DAP-R) score (94.41 before and 92.56 after, with significant difference). The knowledge indirectly affected nursing students’ willingness to organ donation through attitude. Knowledge had a direct and positive impact on attitudes (*β* = 1.564). Additionally, nursing students’ attitudes positively affected their willingness (*β* = 0.023). Attitudes played a mediating role in the relationship between knowledge and willingness (*β* = 0.035). Additionally, attitude toward death, fear of death, and acceptance of the concept of escape were found to be correlated with their willingness.

**Conclusion:**

Organ donation willingness was found to be low among nursing students. Positive attitudes were identified as a mediating factor between knowledge and willingness. Additionally, DAP-R was a related factor. Therefore, it is recommended to focus on improving knowledge and attitude, as well as providing death education to help nursing students establish a positive attitude toward death. These efforts can contribute to the promotion of organ donation.

## 1 Background

Transplantation is considered the best and the only life-saving treatment for end-stage organ failure (1). In recent years, there has been a global increase in the demand for organ donation due to the rising incidence of organ failure (2). Organ donation can be defined as the legal process of removing and transferring biological tissue or organs from a living person, with their consent, or from a deceased person, with the consent of their next of kin, to a recipient in need of transplantation (2). According to the most recent data from the WHO Global Observatory on Donation and Transplantation, over 130,000 solid organ transplants are performed worldwide (1). However, it is estimated that this number represents less than 10% of the global need (3). Survey data from 2021 shows that the number of donations per million people in the United States and Spain was 41.88 and 40.2, respectively, while in China, the number was only 3.63 people (4).

Knowledge and attitudes toward organ donations have a direct impact on the willingness to donate an organ (5). Traditional cultural beliefs, such as the idea that “our body, hair, and skin are given by our parents” and the notion of keeping the body intact after death, have had a significant influence on people’s attitudes toward organ donation. The topic of death is often avoided, and there are many concerns and reservations surrounding organ donation. These sociocultural factors play a role in shaping individuals’ attitudes toward donation (6).

Medical students represent a vital resource in promoting the development of organ donation. Their attitudes toward donation are instrumental in raising public awareness and fostering positive attitudes and willingness to donate organs (7). According to the Medical Council of Canada, graduating medical students should possess the skills to activate appropriate organ donation protocols in cases of brain death (8). This includes counseling the family about the possibility of organ donation (8). Unfortunately, inadequate knowledge about organ donation has been observed among health professionals and medical students worldwide (9–13). The future reserve force for clinical workers will be nursing students. Nursing students, present the trainee students undergoing their nursing training, and transferring theoretical knowledge into clinical internship (14, 15). If they develop a positive attitude toward death and a willingness to donate organs during their internship, it can greatly contribute to the identification of potential organ donors in their future work and even encourage them to become potential donors themselves. This, in turn, will significantly promote the development of organ transplantation in China. However, few studies have explored the potential role of attitude in the relationship between knowledge and willingness toward donation among nursing students.

Currently, research both domestically and internationally primarily centers on examining the knowledge, attitudes, and willingness regarding organ donation among nursing students in educational institutions and registered nurses. Researchers also analyze the factors influencing organ donation within hospitals (16–23). However, there are few researches on nursing students in the internship stage, and lack of investigate the mediating role of attitude in the relationship between knowledge and willingness.

The study aims to investigate the levels of knowledge, attitudes, willingness to donate organs, and DAP-R among nursing students and examine whether attitude was a mediator factor of knowledge and willingness. Our goal was to seek to identify factors that may influence the willingness, to promote organ donation and reduce organ shortage.

## 2 Methods

### 2.1 Research design and participants

This was a cross-sectional descriptive study conducted at a hospital located in Wuhan, China, which offers a wide range of medical services and specialties, including but not limited to cardiology, neurology, oncology, pediatrics, orthopedics, and transplant surgery. A cross-sectional, descriptive study was undertaken, utilizing a convenience sampling method to enroll 758 nursing students (*n* = 435/323) before and after their clinical internship. The study was carried out for 3 months from March to May 2022 among before clinical internship nursing students, and from Jan to March 2023 among after clinical practice nursing students. Participants were drawn from 23 medical colleges in Hubei province, China. Inclusion criteria required participants to be full-time nursing students who: (1) were in their final year of study and (2) had completed a minimum of 8 months of clinical internship. (3) Informed consent was obtained regarding the purpose of the study. The exclusion criteria included those who did not complete all the questionnaires for various reasons. Based on the analysis proposed by Burns and Grove (24), each variable required at least 10 participants. 65 variables are included in this study, thus, the estimated sample size was 10 × 65 = 650. All participants were in the same grade and have served same amount of time for their internship. The participants were invited using a software called SoJump. The data were collected using the software backstage. The participants were assured of confidentiality, and the data collected would be used solely for scientific investigation.

### 2.2 Instruments

#### 2.2.1 Demographic characteristics of the sample

The demographic variables of nursing students included age, gender, ethnicity, family residence, income, educational level, political status, and religious belief.

#### 2.2.2 Knowledge, attitude, and willingness to organ donation

Nursing students’ knowledge, attitude, and willingness to organ donation were measured by using the organ donation knowledge scale, the scale developed by Shi Lizhu from Taiwan in 1998 (25). The scale has been applied to several studies involving nursing students and nurse (26, 27).

The organ donation knowledge consists of 10 judgment questions. One point is awarded for each correct answer, while a wrong answer is scored as 0 points. The maximum score one can achieve is 10 points, with a higher score indicating a better level of organ donation knowledge.

The organ donation attitude comprises 22 questions, organized into three dimensions. Dimension 1, “Reasons for obstructing organ donation,” includes questions 12 to 22. Dimension 2, “Agree with the value of organ donation,” covers questions 1 to 6. Dimension 3, “Disagree with the value of organ donation,” encompasses questions 7 to 8 and 10 to 11. The scale is scored on a 7-point Likert scale, ranging from “very disagree” (1 point) to “very agree” (7 points). The maximum score achievable is 7 points. In the positive title section (questions 1 to 6), a higher score indicates a more positive attitude toward organ donation. Conversely, in the reverse title section (questions 7 to 22), a higher score indicates a more negative attitude toward organ donation. To facilitate data analysis, the positive questions will be scored in reverse. The total score ranges from 22 to 154, with higher scores indicating a more positive attitude toward organ donation. To facilitate understanding and comparison, we normalized the raw scores, with the transformed score = raw score/22(total questions number of questionnaires).

The organ donation willingness consists of 5 questions. Question 1 assesses personal willingness to donate organs the Likert 5 scoring method. “Unwilling” is scored as 1 point, “thought but has not yet decided” as 2 points, “considered and discussed with the family” as 3 points, “willing but not signed the donation consent card” as 4 points, and “willing and signed the organ donation card” as 5 points. The maximum score one can achieve is 5 points, a higher score indicates a stronger willingness to donate. Question 2 evaluates the willingness to donate organs within the family. Question 3 assesses the willingness to donate organs to others. Question 4 explores the willingness to select the recipient of the donated organ. Lastly, question 5 examines the source of the opinion regarding signing the organ donation card. Questions 1 and 2 are single-choice, while questions 3, 4, and 5 are a multiple-choice.

#### 2.2.3 Dead attitude investigation

The Death Attitude Profile—Revised (DAP-R) is the Chinese edition of the DAP-R developed by Tang Lu and others (27). It consists of 32 items distributed across 5 dimensions: fear of death (items 1, 2, 7, 18, 20, 21, 32), death avoidance (items 3, 10, 12, 19, 26), natural acceptance of death (items 6, 14, 17, 24, 30), approach acceptance of death (items 4, 8, 13, 15, 16, 22, 25, 27, 28, 31), and escape acceptance of death (items 5, 9, 11, 23, 29). Each item is scored on a Likert 5 scale, ranging from “very disagree” to “very agree,” with scores ranging from 1 to 5. The total score ranges from 32 to 160, a higher score in each dimension indicates a greater inclination toward that particular attitude toward death.

### 2.3 Ethical considerations

The study received approval from the Institutional Review Board of Zhongnan Hospital of Wuhan University (NO: 2022124K). Before administering the questionnaire survey, written informed consent was obtained from each participant.

### 2.4 Data analysis

The data were analyzed using Statistical Package for the Social Sciences (SPSS) version 23.0. Demographic characteristics of the study participants were assessed using frequency, percentage, and the Chi-Squared Test. The differences in willingness toward organ donation among nursing students based on sociodemographic characteristics were examined using the Chi-Squared Test. Scores for knowledge, attitude, willingness toward organ donation, and attitudes toward death were analyzed using mean and standard deviation (SD). The Mann–Whitney U test was employed to investigate differences in scores between nursing students before and after practical training, as well as differences in scores between those with and without internship experience in the organ donation department. Spearman correlation coefficients were calculated to evaluate the relationships between attitudes toward death and willingness to donate organs. Additionally, we explored potential mediation effects in the relationship between knowledge, attitude, and willingness using RStudio version 2022.07.1.

## 3 Results

### 3.1 Participants’ characteristics

Initially, a sample of 775 nursing students participated in the study. However, 17 questionnaires were incomplete and were excluded from the final analysis. Therefore, the final survey included 758 nursing students, resulting in a response rate of 97.81%.

The sociodemographic characteristics of the participants are presented in Table 1. The majority of nursing students were female (84.56%), with a mean age of 20.61 years (SD = 1.10). Out of the total participants, 728 (96.04%) identified as Han Chinese. In terms of residency, 421 (55.54%) nursing students’ families lived in cities. Furthermore, 553 (72.96%) nursing students’ families had a monthly income below 695$. Regarding educational background, 347 (45.78%) nursing students held a bachelor’s degree. Only 8.84% of participants were Party Members, and a mere 2.24% reported having religious beliefs (Table 1).

**Table 1:** Sociodemographic characteristics of the participants (n = 758).

Variables	n	%	*X²*	*P*
Age	20.61 (Mean)	1.10 (SD)		
Gender				
Male	117	15.44	0.614	0.433
Female	641	84.56		
Ethnicity				
Han	728	96.04	0.210	0.647
Other minorities	30	3.96		
Families reside				
City	421	55.54	0.043	0.837
Village	337	44.46		
Income				
<139$	52	6.86	0.170	0.680
139$-278$	107	14.12		
279$-417$	157	20.71		
418$-495$	237	31.27		
496$-1389$	156	20.58		
>1389$	49	6.46		
Education level				
Regular Specialized Secondary Schools	14	1.85	0.581	0.446
Short-cycle Courses	397	52.38		
Undergraduate	347	45.78		
Politics status				
Mass	86	11.35	2.197	0.138
League member	605	79.82		
Party Members	67	8.84		
Religious belief				
Yes	17	2.24	0.014	0.907
No	741	97.76		

Regardless of the sociodemographic subgroup, it was found that more than 85% of the respondents were either unwilling or uncertain about donating organs, with only a few exceptions. The analyses revealed that age, residence of families, experience in holding student cadre positions during college, awareness of organ donation methods, and personal or family history of organ transplantation or donation were identified as independent factors influencing willingness to donate organs (*p* < 0.05). Only 5.56% of the nursing students over the age of 21 would donate organs, whereas 12.05% among those under 21 would donate organs. The nursing students’ reside from city had the higher willingness, with 14.25%, while only 6.53% of nursing students living in village would donate organs. Only 12.97% of nursing students who had ever served as a student cadre during college would donate organs, if the nursing students who had not ever served as a student cadre during college, only 8.08% would donate organs. If they aware of the organ donation from relatives and friends cases around them, 19.58% of nursing students would donate organs. 44.83% of nursing students who had signed organ donation consent cards would donate organs, while 55.17% were not. On the other hand, factors such as gender, ethnicity, income, education level, political status, religion, experience in welfare activities, and having family members who are medical personnel did not show a significant correlation (*p* > 0.05, Table 2).

**Table 2:** Sociodemographic distribution and willingness in organ donation of participants (*n* = 758).

Variables		Willingness in organ donation			
		Yes	No/not sure		
	*n*(%)	*n*(%)	*n*(%)	*x* ^2^	*p*
Age					
≤21	614(81.00)	74(12.05)	540(87.95)	5.103	0.024
>21	144(19.00)	8(5.56)	136(94.44)		
Gender					
Male	117(15.44)	16(13.68)	101(86.32)	1.171	0.297
Female	641(84.56)	66(10.30)	575(89.70)		
Ethnicity					
Han	728(96.04)	81(11.13)	647(88.87)	1.814	0.295
Other minorities	30(3.96)	1(3.33)	29(96.67)		
Families reside					
City	421(55.54)	60(14.25)	361(85.75)	11.573	0.001
Village	337(44.46)	22(6.53)	315(93.47)		
Income					
<139$	52(6.86)	3(5.77)	49(94.23)	3.464	0.629
139$-278$	107(14.12)	10(9.35)	97(90.65)		
279$-417$	157(20.71)	16(10.19)	141(89.81)		
418$-495$	237(31.27)	28(11.81)	209(88.19)		
496$-1389$	156(20.58)	17(10.90)	139(89.10)		
>1389$	49(6.46)	8(16.33)	41(83.67)		
Education level					
Secondary	14(1.85)	0(0)	14(100)	2.012	0.172
Tertiary	397(52.37)	46(11.59)	351(88.41)		
Undergraduate	347(45.78)	36(10.37)	311(89.63)		
Politics status					
Mass	86(11.35)	13(15.12)	73(84.88)	5.949	0.051
League member	605(79.82)	67(11.07)	538(88.93)		
Party Members	67(8.84)	2(2.99)	65(97.01)		
Religious belief					
Yes	17(2.24)	2(11.77)	15(88.24)	0.016	1
No	741(97.76)	80(10.80)	661(89.20)		
Have you ever served as a student cadre during technical secondary school or university
	Yes	424(55.94)	55(12.97)	369(87.03)	4.627	0.031
	No	334(44.06)	27(8.08)	307(91.92)		
	Experience in welfare activities					
	Yes	714(94.20)	78(10.92)	636(89.08)	0.016	1
	No	44(5.81)	4(9.09)	40(90.91)		
Family members are medical personnel					
Yes	445(58.71)	56(12.58)	389(87.42)	3.485	0.062
No	313(41.29)	26(8.31)	287(91.69)		
Aware of the Ways to Donate Organs					
Social propaganda	570 (75.20)	61 (10.70)	509 (89.30)	14.573	0.026
School publicity	381(50.26)	40(10.50)	341(89.50)		
Hospital promotion	440(58.05)	60(13.64)	380(86.36)		
Introduction to family/friends	119(15.70)	16(13.45)	103(86.55)		
Surrounding cases	143(18.87)	28(19.58)	115(80.42)		
Others	36(4.75)	2(5.56)	34(94.44)		
lack of awareness	56(7.39)	3(5.36)	53(94.64)		
Personal or family experience					
Organ donation experience	19(2.51)	6(31.58)	13(68.42)	54.309	<0.001
Experience in organ transplantation	20(2.64)	6(30)	14(70)		
Patients who have previously taken care of organ donors or transplant recipients	65(8.58)	16(24.62)	49(75.38)		
Received education related to organ donation	192(25.33)	31(16.15)	161(83.85)		
Promoted or promoted organ donation	50(6.60)	11(22)	39(78)		
Sign the organ donation consent card	29(3.83)	13(44.83)	16(55.17)		
Others	13(1.72)	1(7.69)	12(92.31)		
None	500(65.96)	42(8.4)	458(91.6)		

### 3.2 Scores of organ donation cognition and DAP-R

The Cronbach’s alpha coefficients for the organ donation willingness scale were 0.930 in the original study (25) and 0.889 in this study. For the DAP-R, the Cronbach’s alpha coefficients were 0.875 in the original study (28) and 0.865 in this study.

The mean score for knowledge of organ donation before the clinical internship was 7.17 while 7.22 at the end stage of clinical internship with no significant difference between the scores before and after internship (*p* > 0.05). The mean score for attitude toward organ donation before clinical internship was 4.58 whereas 4.36 at the end stage of clinical internship with a significant difference in the scores before and after internship (*p* < 0.001). The mean score for willingness to donate organs before clinical internship was 2.26 whereas 2.07 at the end stage of clinical internship with a significant difference between the scores before and after internship (*p* < 0.001) (Table 3).

**Table 3:** Scores of organ donation cognition.

Variables	Before clinical internship (*n* = 435)		The end stage of clinical internship (*n* = 323)			
	Mean	SD	Mean	SD	*Z*	*p*
Knowledge	7.17	1.08	7.22	1.16	−1.032	0.302
Attitude	4.58	0.81	4.36	0.71	−4.538	<0.001
Willingness	2.26	0.88	2.07	0.87	−3.504	<0.001

The average DAP-R score before the clinical internship was 94.41 ± 14.62, and at the end stage of clinical internship, it was 92.56 ± 14.80. There was no significant difference in scores before and after the clinical internship (*p* > 0.05). The average fear of death to DAP-R score before the clinical internship was 20.47 ± 4.78, and at the end stage of clinical internship, it was 19.56 ± 4.36. There was a significant difference in scores before and after the clinical internship (*p* = 0.002). The average death avoidance to DAP-R score before the clinical internship was 14.40 ± 3.40, and at the end stage of clinical internship, it was 14.01 ± 3.30. There was no significant difference in scores before and after the clinical internship (*p* > 0.05). The average natural acceptance of death to DAP-R score before the clinical internship was 17.48 ± 3.10. At the end stage of clinical internship, the score was 16.81 ± 3.06. There was a significant difference between the scores before and after the clinical internship (*p* = 0.008). The average approaching acceptance of death to DAP-R score before the clinical internship was 28.12 ± 6.08. At the end stage of clinical internship, the score was 28.16 ± 6.00. There was no significant difference between the scores before and after the clinical internship (*p* > 0.05). The average escape acceptance of death to DAP-R score before the clinical internship was 13.94 ± 3.56. At the end stage of clinical internship, the score was 14.02 ± 3.66. There was no significant difference between the scores before and after the clinical internship (*p* > 0.05) (Table 4).

**Table 4:** Scores of DAP-R.

Variables	Before clinical internship (*n* = 435)		The end stage of clinical internship (*n* = 323)			
	Mean	SD	Mean	SD	*Z*	*p*
Scores of nursing students’ DAP-R	94.41	14.62	92.56	14.80	−1.944	0.052
Fear of death	20.47	4.78	19.56	4.36	−3.084	0.002
Death avoidance	14.40	3.40	14.01	3.30	−1.877	0.06
Neutral acceptance of death	17.48	3.10	16.81	3.06	−2.638	0.008
Approach acceptance of death	28.12	6.08	28.16	6.00	−0.129	0.897
Escaping acceptance of death	13.94	3.56	14.02	3.66	−0.359	0.72

DAP-R, Death Attitude Profile-Revised.

The mean score for knowledge of organ donation which have internship experience in the department of organ donation was 7.24 while have not was 7.19, with no significant difference (*p* > 0.05). The mean score for attitude of organ donation which have internship experience in the department of organ donation was 4.37 while have not was 4.34, with no significant difference (*p* > 0.05). The mean score for attitude of organ donation which have internship experience in the department of organ donation was 2.13 while have not was 1.94, with no significant difference (*p* > 0.05) (Table 5).

**Table 5:** Scores of organ donation cognition.

Variables	Internship experience in the Department of organ donation					
	Yes (*n* = 213)		No (*n* = 111)			
	Mean	SD	Mean	SD	*Z*	*p*
Knowledge	7.24	1.11	7.19	1.25	−0.144	0.885
Attitude	4.37	0.72	4.34	0.69	−0.197	0.844
Willingness	2.13	0.94	1.94	0.72	−1.544	0.123

The mean score for DAP-R which have internship experience in the department of organ donation was 91.91 while have not was 93.83, with no significant difference (*p* > 0.05). The mean score for fear of death which have internship experience in the department of organ donation was 19.34 while have not was 19.97, with no significant difference (*p* > 0.05). The mean score for death avoidance which have internship experience in the department of organ donation was 13.86 while have not was 14.30, with no significant difference (*p* > 0.05). The mean score for neutral acceptance of death which have internship experience in the department of organ donation was 16.72 while have not was 16.99, with no significant difference (*p* > 0.05). The mean score for approach acceptance of death which have internship experience in the department of organ donation was 28.13 while have not was 28.23, with no significant difference (*p* > 0.05). The mean score for escaping acceptance of death which have internship experience in the department of organ donation was 13.85 while have not was 14.34, with no significant difference (*p* > 0.05) (Table 6).

**Table 6:** Scores of DAP-R.

Variables	Internship experience in the Department of organ donation					
	Yes (*n* = 213)		No (*n* = 111)			
	Mean	SD	Mean	SD	*Z*	*p*
Scores of nursing students’ DAP-R	91.91	15.09	93.83	14.14	−1.269	0.204
Fear of death	19.34	4.33	19.97	4.39	−0.982	0.326
Death avoidance	13.86	3.42	14.30	3.01	−1.377	0.169
Neutral acceptance of death	16.72	3.04	16.99	3.09	−0.854	0.393
Approach acceptance of death	28.13	5.97	28.23	6.07	−0.696	0.486
Escaping acceptance of death	13.85	3.60	14.34	3.75	−0.595	0.552

DAP-R, Death Attitude Profile-Revised.

### 3.3 Mediation analyses of organ donation willingness

**Figure 1:**
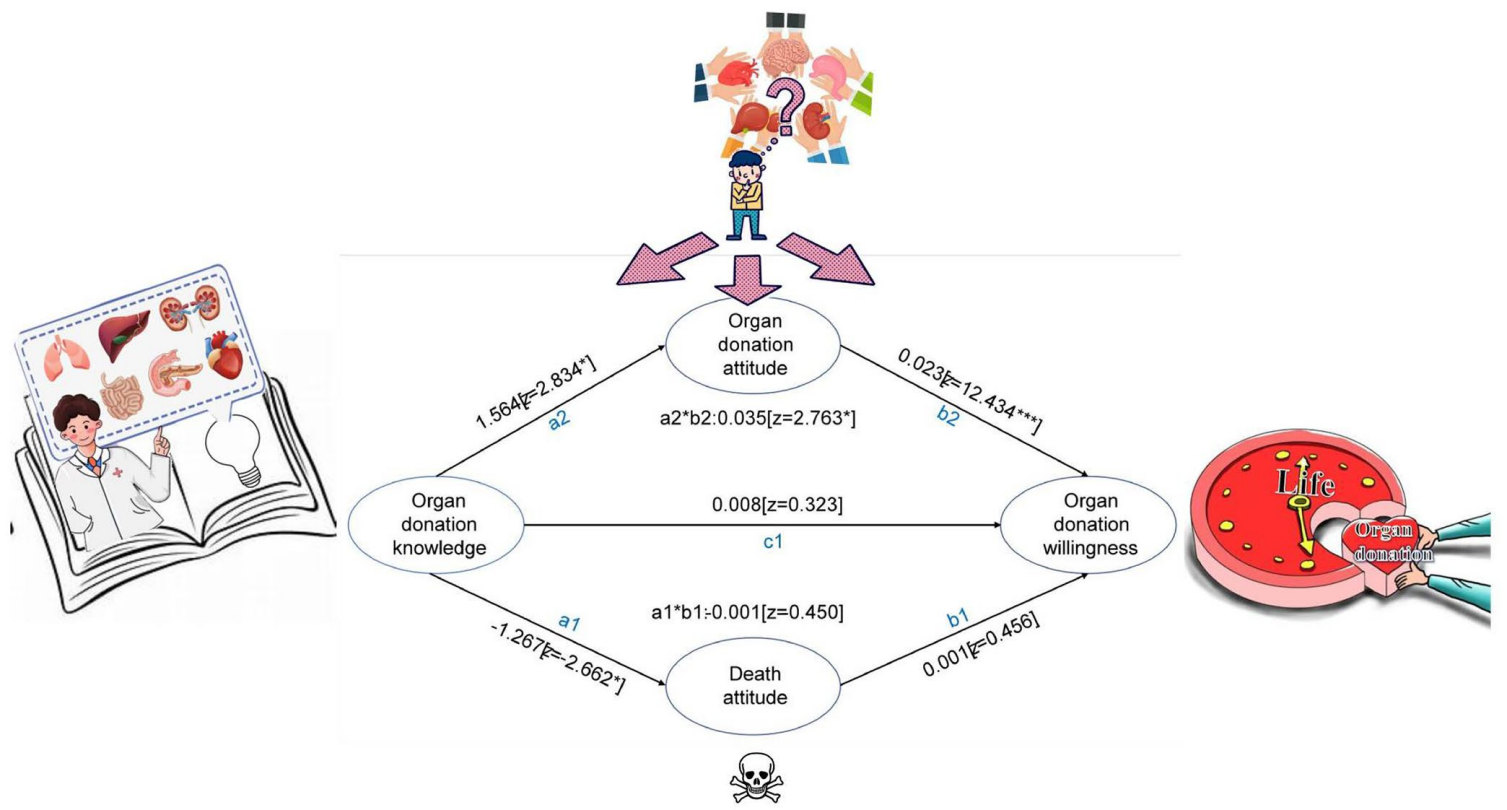
Mediation model of the study.

Mediation analyses revealed that organ donation attitude accounted for a significant proportion of the association between knowledge and willingness. The risk factor that contributed to the relationship is the organ donation attitude of 0.035 (Figure 1). Being ≤21 years old (0.037), families residing in a village (0.036), and having ever served as a student cadre during technical secondary school or university (0.045) (Table 7).

**Table 7:** Mediating effects (a2*b2).

Subgroup	*β*	SD	*p*	CI. lower	CI. upper
All	0.036	0.0128	0.0057	0.0103	0.0606
Age					
≤21	0.037	0.0188	0.0494	0.0001	0.0736
>21	0.034	0.0179	0.0599	-0.0014	0.0687
Families reside					
City	0.032	0.0192	0.1015	-0.0062	0.0692
Village	0.036	0.0157	0.022	0.0052	0.0669
Have you ever served as a student cadre during technical secondary school or university?					
Yes	0.045	0.0172	0.0097	0.0108	0.0783
No	0.021	0.0188	0.2657	-0.0159	0.576

SD, Standard Deviation; CI, Confidence interval.

### 3.4 Correlation between attitude toward death and willingness to organ donation among nursing students

Spearman correlation analysis was conducted to explore the relationship between various dimensions of the DAP-R and willingness toward organ donation. The analysis revealed statistically significant correlations between fear of death, death avoidance, escaping acceptance of death, and the overall DAP-R score with willingness (Table 8). However, the correlations between fear of death, death avoidance, and the DAP-R score were found to be weak and negative, with Spearman’s Rho values of −0.17, −0.111, and − 0.076, respectively (*p* Value <0.05). Conversely, a strong and positive correlation was observed between escaping acceptance of death and willingness, with a Spearman’s Rho of 0.684. Notably, no statistically significant correlations were found between neutral acceptance of death, approach acceptance of death, and willingness (Table 8).

**Table 8:** Correlation between attitude toward death and willingness to organ donation among nursing students.

	Fear of death	Death avoidance	Neutral acceptance of death	Approach acceptance of death	Escaping acceptance of death	DAP-R
Rho	−0.17	−0.111	0.069	−0.011	0.684	−0.076
*p*	<0.001	0.002	0.056	0.757	<0.001	0.036

Death Attitude Profile-Revised (DAP-R).

### 3.5 Reasons for unwillingness to donate organs

Through this study, seven reasons were identified as to why nursing students are unwilling to donate organs. The most significant reason is that the majority of nursing students are not familiar with the donation process (*n* = 293/758; 38.65%). Additionally, many nursing students are influenced by traditional concepts (*n* = 274, 36.15%) or have concerns about potentially harming their interests (*n* = 194, 25.59%). A considerable number of nursing students (*n* = 165, 21.77%) worry about incomplete laws related to organ donation. Some nursing students are concerned about high costs (*n* = 109, 14.38%) or perceive the organ donation process as complex (*n* = 102, 13.46%). The remaining reasons fall into the category of “other” (*n* = 71, 9.37%).

## 4 Discussion

Organ transplantation supports save patients’ life and improves their survival rates (29). However, there can be no organ transplant without organ donation. Nurses played an important role in guiding organ donation decisions in many global health settings (30). Good knowledge and a high attitude are critical in promoting organ donation and transplantation (31). Therefore, as the reserve army of the nursing team, the cognition and attitude of nursing students toward organ donation are very important. Organ transplantation supports save patients’ life and improves their survival rates (29). Therefore, this study aims to assess nursing students’ knowledge, attitudes, willingness toward organ donation and DAP-R, and seeks to identify factors that may influence the willingness. Finally, focuses on the relationships among DAP-R, organ donation knowledge, attitude, and willingness (16).

The knowledge of organ donation among nursing students in our study was found to be at a medium level. This result is consistent with previous studies conducted in both China and Western countries (27, 32), highlighting that nursing students’ understanding of organ donation is not comprehensive enough. Notably, the items with the highest error rates in both the before and after clinical internship answers of nursing students are related to organ donation legal knowledge. This suggests that despite their medical background and a higher level of knowledge in organ transplantation and donation compared to non-medical students (33), nursing students still lack knowledge of relevant laws and regulations (34). To address this gap, medical colleges can consider incorporating targeted courses on laws and regulations related to organ donation into their professional curriculum, to enhance nursing students’ understanding of organ donation comprehensively. Additionally, it is worth noting that 25.59% of nursing students provided incorrect answers regarding the concept of brain death, which is similar to the level observed in foreign countries (35). This indicates that there is a need for greater efforts in disseminating relevant knowledge in China, similar to the level of awareness abroad.

The main factors that influence the attitude of nursing interns toward organ donation are the hindrances to organ donation, agreement with the value of organ donation, and disagreement with the value of organ donation. The studies conducted by Lei et al. (27), highlight that nursing students generally hold a positive attitude toward organ donation. The scores across various dimensions indicate that nursing students also tend to identify with the value of organ donation. The main reasons cited for hindering organ donation include concerns about incomplete corpses, traditional Chinese concepts of death, and fears that organs may be transplanted to unworthy recipients, thereby harming society. These reasons may be attributed to traditional Chinese beliefs, as the prevailing notion is that organs should not be removed after death due to cultural taboos. Consequently, the concept of organ donation is not widely embraced in China. To improve the attitude of nursing students toward organ donation, it may be beneficial to incorporate lectures on organ donation in educational institutions and hospitals.

Despite the relatively positive attitude toward organ donation among our nursing students, the willingness to donate organs remains low. In the current study, only 10.818% of the nursing students expressed willingness to donate, and out of those, only 13 had signed the organ donation form. This donation rate is much lower compared to many developed countries such as Japan (43.6%), Poland (73.6%), and Italy (85.0%) (27). While the factors contributing to the low organ donation rate among Chinese individuals may be diverse, our study through One-way Anova analyses indicate that age, family residence, experience as a student cadre during college, awareness of organ donation methods, and personal or family experience with organ donation or transplantation may independently influence willingness to donate organs. Nevertheless, it is evident that early education on organ donation for nursing students, through exposure to relevant cases, is of critical importance in fostering a higher willingness to donate organs.

A study conducted in Iran found that medical students’ unwillingness to become organ donors was primarily influenced by their religious beliefs and concerns about the integrity of their bodies being disrupted (36). In Egypt, the reluctance to donate organs was attributed to a lack of trust in a highly commercialized healthcare system (37). In our study, we found that the primary obstacle to organ donation among nursing students is a lack of knowledge about the organ donation process. This is consistent with other studies (1). The second most frequently cited reason is the influence of traditional beliefs, followed by concerns about potential harm to one’s interests and inadequate organ donation laws. Therefore, it is necessary to further enhance publicity and education for nursing students, enabling them to understand that their remains will be genuinely utilized and respected. This will help alleviate concerns and encourage voluntary donations. At the same time, we should continue to enhance laws and regulations about organ donation, streamline the donation procedure, and enhance the oversight mechanism to provide donors with a sense of security and comfort. This will help alleviate any concerns donors may have and safeguard their rights and interests.

The results of this study indicate that there is no statistically significant difference in the knowledge of organ donation among nursing students before and after clinical internship. However, there are statistically significant differences in their attitudes and behaviors toward organ donation. After the clinical internship, it was noted that the attitude and behavior scores of nursing students decreased compared to before. We hypothesize that this decrease may be attributed to the high work pressure experienced by nursing students after completing their internship. Through the research, we found that the nursing students after the internship had a lower fear of death, but this was not related to whether they had internship experience in the organ donation department. People who avoid talking about death in their daily lives will have fear and avoidance of death, thereby reducing the sources of organ donation, and ultimately hindering the development of organ donation (38).

The Spearman’s correlation analysis of organ donation willingness shows that escape acceptance of death is one of the main factors affecting the organ donation willingness of college students, and increasing escape acceptance of death will promote organ donation willingness. Other research indicated fear of death, death avoidance, approach acceptance of death, and escape acceptance of death emerged as independent factors that might influence willingness in organ donation (27). As future caretakers of patients in their final moments, nursing students hold a significant role. Furthermore, a healthy understanding of life and death equips them to provide positive guidance to dying patients and their families. By approaching death with a balanced perspective, they can help individuals view it in a more rational light. This, in turn, may enhance their willingness to donate organs.

Our study showed that there had no effect between the scores of organ donation cognition and DAP-R internship experience in the department of organ donation. Since there are few researches in related fields, we speculate that the reason for this situation is that clinical learning in China pays more attention to the training of clinical skills (39). China has been carrying out organ donation after the death of citizens for 10 years. Currently, 188 hospitals are qualified for organ transplantation (40), and the courses for clinical learning in transplant departments are not perfect. In China, there have attach importance to practical skills training and neglect organ donation knowledge education in clinical learning. Therefore, we believe that in the clinical learning of interns, especially hospitals with organ transplantation qualifications should take advantage of their own advantages to carry out education and publicity of relevant contents for nursing students.

Our study focuses on the relationship between knowledge, attitude, and willingness regarding organ donation. The results suggest that knowledge of organ donation does not directly impact the organ donation willingness of nursing students, but organ donation attitude can indirectly affect the willingness to organ donation. Therefore, it is crucial to find ways to improve nursing students’ attitudes toward organ donation. It has been reported that in countries like Denmark and Saudi Arabia, some people reject organ donation due to religious reasons, as they believe in preserving the integrity of the body (38, 41). In China, people often prioritize preserving the integrity of the body due to Confucian teachings on filial duty and the belief in a second life after death in another world (42). Although most nursing students have certain knowledge and positive attitudes toward organ donation, their willingness still needs to be improved. This implies the need for intensified relevant education to raise their knowledge and attitude and increase their willingness to donate organs.

## 5 Limitation of this study

This study has certain limitations that need to be acknowledged. Firstly, it should be noted that the majority of participants in this study are from the same province, which may restrict the generalizability of the findings to a larger population of nursing students. Secondly, the results of this study heavily rely on self-reports from nursing students, which introduces the possibility of socially desirable responses. Thirdly, despite assuring the nursing students that the survey was anonymous, there is still a chance that they may withhold honest thoughts. Lastly, it is important to notify the features of the participants in this study. The mean age of our participants was 20.61 years old. Given their young age, these nursing students may lack the necessary experience and maturity in dealing with organ donation issues. This lack of experience could potentially impact their attitudes and willingness toward organ donation to some extent. Meanwhile, more females than males were recruited in this study, which may influence the generalizability of our results. It has been reported that female gender was statistically significant in prediction of positive attitudes toward organ donation (43). A recent study also showed that women were more willing to donate their organs to family members and strangers compared with men (44). Therefore, eliminating gender imbalance will produce greater generalizability of study.

## 6 Conclusion

In our current study, it was found that nursing students generally possessed a better understanding of organ donation knowledge, had a more positive attitude toward organ donation and death, and were more willing to donate organs compared to the general Chinese public. However, it is important to note that the overall willingness to donate organs was still low, primarily due to the influence of family-centered traditional values and attitudes toward death. To address this issue, it is recommended that targeted education and training programs be implemented to enhance organ donation-related knowledge and foster positive attitudes toward death among nursing students and the general public. These initiatives are crucial for motivating individuals and maintaining a high level of willingness toward organ donation.

## Data availability statement

The original contributions presented in the study are included in the article/supplementary material, further inquiries can be directed to the corresponding authors.

## Ethics statement

The studies involving humans were approved by the study received approval from the Institutional Review Board of Zhongnan Hospital of Wuhan University (NO: 2022124K). The studies were conducted in accordance with the local legislation and institutional requirements. The participants provided their written informed consent to participate in this study.

## Author contributions

XC: Data curation, Formal analysis, Writing – original draft. XZ: Data curation, Methodology, Writing – original draft, Writing – review & editing. YX: Data curation, Formal analysis, Writing – review & editing. LP: Data curation, Methodology, Writing – review & editing. PL: Data curation, Formal analysis, Writing – review & editing. WL: Formal analysis, Methodology, Writing – review & editing. LJ: Formal analysis, Supervision, Writing – review & editing. CZ: Formal analysis, Methodology, Writing – review & editing. CH: Data curation, Validation, Writing – review & editing.
